# Comparison of the quadratus lumborum versus erector spinae block for postoperative analgesia in hip surgery

**DOI:** 10.6026/973206300212311

**Published:** 2025-08-31

**Authors:** Santosh Kharadi, Arvind Kumar Rathiya, Kuldeep Kumar Patel

**Affiliations:** 1 Department of Anaesthesiology, Government Medical College Ratlam Madhya Pradesh, India; 2 Department of Anaesthesiology, S.S. Medical College & Associated Hospitals, Rewa Madhya Pradesh, India

**Keywords:** Pain, hip surgery, regional anesthesia

## Abstract

Regional anesthetic techniques help alleviate postoperative pain, decrease the reliance on narcotic analgesics and reduce the negative side effects linked to opioids in patients undergoing hip surgery. Orthopedic procedures, including hip and femur fracture surgeries can result in considerable postoperative discomfort. Therefore, it is of interest to assess the efficacy of the Quadratus Lumborum Tran muscular block and the Erector Spinae block at the L4 level in delivering pain relief after hip surgery. Data shows that both Quadratus Lumborum and Erector Spinae Plane blocks are effective for postoperative analgesia in hip surgery. These techniques also enhance the quality of multimodal analgesia compared to the control group.

## Background:

Post-operative pain management is a crucial component of Enhanced Recovery after Surgery (ERAS) protocols for elective surgeries. However, the introduction of regional analgesia has provided alternatives to systemic opioids [1]. Side effects of opioids-such as sedation, respiratory depression, constipation and delayed patient mobilization-have prompted anesthetists to seek ways to reduce opioid use. Spinal or Epidural anesthesia during major hip surgery has been associated with reduced perioperative complications, such as deep venous thrombosis and pulmonary complication in high-risk patients and decreased blood loss [2]. The Erector Spinae Plane (ESP) block, a relatively recent technique for analgesia, was first depicted by Forero *et al.* in 2016 for treating thoracic neuropathic pain [3]. Erector Spinae block (ESP) has been used in both adults and children for various indications, including chronic shoulder pain (T2), thoracic and breast surgery (T4-5) and upper abdominal surgery (T7-8). .The ESP block is typically done at T5-T7 paraspinal levels but can also be performed at the lumbar level and its application has expanded to include postoperative analgesia for a wide range of surgeries, from shoulder to hip procedures [4]. The risk of complications like hematoma formation in USG-guided L-ESPB is relatively low due to the lack of direct blood vessel contact, eliminating the risk of mechanical nerve damage. QLB (Quadratus Lumborum) has been employed to reduce postoperative pain following cesarean sections, laparotomies, laparoscopic procedures and hip surgeries [5]. The primary advantage of QLB over the transverse abdominis plane block is its ability to extend local anesthetic diffusion beyond the transverse abdominis plane to the thoracic paravertebral region, resulting in a broader analgesic effect and longer duration of action [6]. Therefore it is of intrest to show efficacy of Quadratus lumborum tranmuscular (QL-T) and Erector spinae blockat L4 level (L-ESB) for postoperative analgesia in hip surgery.

## Methodology:

This prospective study involved 90 patients from the Department of Anesthesiology at Shyam Shah Medical College & Associated Sanjay Gandhi Memorial Hospital in Rewa. The research was conducted from September 1, 2022, to August 30, 2023. Following approval from the Institutional Ethics Committee (IEC/MC/2022 20902, Date: 12/09/2022), a comprehensive medical history was collected from all selected patients. Those with pre-existing conditions such as diabetes and hypertension were excluded from the study. A thorough pre-anesthetic evaluation, including airway and block site assessments, was conducted. Written informed consent was obtained from patients regarding the surgery, anesthesia and their participation in the study. Randomization into three groups (Group Q, E and C) was achieved using computer-generated tables. To eliminate bias, a double-blind method was employed throughout the study. All blocks were performed by a single investigator post-randomization and both participants and outcome assessors were blinded to group assignments. To ensure participant blinding, all blocks were executed after the surgical procedure. A trained staff nurse, unaware of the specific procedures, conducted the assessments. Statistical analysis was performed with assistance from a statistician. Patients were informed about the entire procedure in a comprehensible manner and educated on the Numeric Rating Scale (NRS) and patient satisfaction metrics. After data collection, the information was entered into Microsoft Office Excel and analyzed using EpiInfo7, free software.

## Results:

Out of total 90 patients each group had 30 patients. The mean age in Group Q, E, C was 55.36 ± 14.11 years, 49.03 ± 13.21years & 48.33 ± 10.37 years. The mean body weight was 59.56 ±4.87kg, 56.80 ± 7.27 kg and 56.50 ± 6.25 kg for patients of Group Q, Group E and Group C respectively. Proportion of female was 60% in group Q, 36.7% in group E and 23.3% in group C similarly the proportion of male was 40% in Group Q, 63.3% in Group E and 76.7% in Group C. After 1 hr of surgery, NRS was 0.15 ± 0.60 in patients of Group Q, 0.13 ± 0.55 inpatients of Group E and 4.12 ± 1.20 in patients of Group C. NRS was higher in Group Compared to Group Q and Group E which was statistically significant (p-value<0.05). However there was no statistically significant difference observed between Group Q and Group E (p value-0.530). After 2 hrs of surgery, NRS was 1.80 ± 0.47 in patients of Group Q, 1.83±0.87 inpatients of Group E and 3.16 ± 0.64 in patients of Group C. NRS was higher in Group Compared to Group Q and Group E which was statistically significant (p value<0.05). However there was no significant difference between Group Q and Group E (p value-0.546). After 4 hrs of surgery, NRS was 2.73 ± 0.91 in patients of Group Q, 1.83 ± 0.84 in patients of Group E and 3.43 ± 0.72 in patients of Group C. NRS was higher in Group Compared to Group Q and Group E which was statistically significant (P value <0.05). However there was no statistically significant difference observed between Group Q and Group E (pvalue-0.270). After 6 hrs of surgery, NRS was 2.83 ± 1.18 in patients of Group Q, 2.96 ± 0.64 inpatients of Group E and 4.63 ± 0.66 in patients of Group C.

NRS was higher in Group Compared to Group Q and Group E which was statistically significant (p value<0.05). However there was no statistically significant difference observed between Group Q and Group E (p value- 0.502). After 8 hrs of surgery, NRS was 2.66 ± 1.06 in patients of Group Q, 2.73 ± 0.26 inpatients of Group E and 4.56 ± 0.89 in patients of Group C. NRS was higher in Group C compared to Group Q and Group E which was statistically significant (p value<0.05). However there was no statistically significant difference observed between Group Q and Group E (p value-0.186). After 12 hrs of surgery, NRS was 2.90 ± 0.71 in patients of Group Q, 2.80 ± 0.61 in patients of Group E and 4.33 ± 0.84 in patients of Group C. NRS was higher in Group C compared to Group Q and Group E which was statistically significant (P value<0.05). However there was no significant difference between Group Q and Group E (p value-0.603). After 24 hrs of surgery, NRS was (3.73 ± 0.91) in patients of Group Q, (3.57 ± 0.82) inpatients of Group E and (4.77 ± 0.90) in patients of Group C. NRS was higher in Group compared to Group Q and Group E which was statistically significant (p value<0.05). However there was no statistically significant difference observed between Group Q and Group E (p value-0.458) as shown in Table 1 (see PDF).

## Discussion:

Various regional anesthesia techniques are utilized as part of multimodal analgesia in hip surgeries [7]. While epidural analgesia is the gold standard, other effective options include quadratus lumborum (QL), psoas compartment block, paravertebral block and transverse abdominal plane block LA administered in QLB and psoas compartment block spreads to the lumbar plexus, providing analgesia. The risk of complications like hematoma formation in USG-guided ESB-L is relatively low due to the lack of direct contact of blood vessels, eliminating the risk of mechanical nerve damage [8]. Though ESB is a relatively new technique with an unclear mechanism of action, recent studies suggest it as an alternative when lumbar plexus block (LPB) cannot be performed or fails. QLB-T is a newly popular peripheral nerve block with limited case reports and clinical study on its use in hip surgery multimodal analgesia [9]. Both blocks significantly reduce NRS pain scores in the first postoperative hours and decrease analgesic requirements within the first 24 hours compared to standard IV analgesia and low pain scores and analgesia requirements within the first 24 hours postoperatively in both block groups, indicating that L-ESB and QLB-T are effective analgesic methods [10].

## Conclusion:

Data shows that both Quadratus Lumborum and Erector Spinae Plane blocks are effective for postoperative analgesia in hip surgery. These techniques also enhance the quality of multimodal analgesia compared to the control group.
